# Mechanism of action of Coptidis Rhizome in treating periodontitis based on network pharmacology and in vitro validation

**DOI:** 10.1186/s12903-024-04311-9

**Published:** 2024-05-04

**Authors:** Wei Li, Ruofeng Jiao, Shiyi Luo, Zefei Liu, Jukun Song, Zhu Chen

**Affiliations:** 1 Guiyang Hospital of Stomatology, Guiyang, Guizhou 550005 China; 2 Zunyi Medical University, Zunyi, Guizhou 563000 China; 3 Medical College of Guizhou University, Guiyang, Guizhou 550025 China; 4 Department of Oral and Maxillofacial Surgery, The Affiliated Stomatological Hospital of Guizhou Medical University, Guiyang, 550001 China

**Keywords:** Network pharmacology, Coptidis Rhizome, Periodontitis, Berberine, IL-17 Signaling pathway, Molecular docking, In vitro experimental verification

## Abstract

**Objective:**

Explore the therapeutic mechanism of Coptidis Rhizome (CR) in periodontitis using network pharmacology, and validate it through molecular docking and in vitro experiments.

**Methods:**

Screened potential active components and target genes of CR from TCMSP and Swiss databases. Identified periodontitis-related target genes using GeneCards. Found common target genes using Venny. Conducted GO and KEGG pathway analysis. Performed molecular docking and in vitro experiments using Berberine, the main active component of CR, on lymphocytes from healthy and periodontitis patients. Assessed effects on inflammatory factors using CCK-8, flow cytometry, and ELISA.

**Results:**

Fourteen active components and 291 targets of CR were identified. 30 intersecting target genes with periodontitis were found. GO and KEGG analysis revealed oxidative stress response and IL-17 signaling pathway as key mechanisms. Molecular docking showed strong binding of Berberine with ALOX5, AKT1, NOS2, and TNF. In vitro experiments have demonstrated the ability of berberine to inhibit the expression of Th17 + and other immune related cells in LPS stimulated lymphocytes, and reduce the secretion of IL-6, IL-8, and IL-17.

**Conclusion:**

CR treats periodontitis through a multi-component, multi-target, and multi-pathway approach. Berberine, its key component, acts through the IL-17 signaling pathway to exert anti-inflammatory effects.

## Introduction

Coptidis Rhizome (CR), known as Huanglian in Chinese, is mainly used in Asian countries as a traditional Chinese medicine and has been used to treat various inflammatory diseases for a thousand years [1]. In recent years, it has been found that CR also has antibacterial [2], antiviral [3] and anticancer pharmacological effects [4, 5]. More than 100 chemical components were isolated and identified from CR, mainly including berberine, flavonoids, and lignin [1]. Among them, berberine (BBR) is the most representative and abundant one [1]. To explore its mechanism of action in treating these diseases, the researchers conducted a series of mouse experiments, in vitro cell experiments, and network pharmacology analysis on CR. Published studies have found that CR plays a key pharmacological role in type 2 diabetes [6], Alzheimer's disease [7], nonalcoholic fatty liver disease-related liver cancer [8] and other diseases [9].

Periodontitis, considered the sixth most prevalent disease in the world [10], is a chronic infectious inflammatory disease characterized by loss of clinical attachment and destruction of the supporting tissues of the teeth. At present, in addition to conventional systemic therapy, there is also laser therapy for the treatment of oral diseases, such as the surgical method of laser treatment for oral craniofacial tumors studied by Afrah A Aldelaimi et al. [11, 12]. Because periodontitis is mainly caused by bacterial infections, antibiotics are usually used as adjunctive drugs after treatment, but excessive use of antibiotics leads to a surge in bacterial resistance [13]. The emergence and spread of antibiotic resistance genes pose a serious threat to human and animal health worldwide, with infections caused by antibiotic resistant bacteria being the main public health threat [14]. Scholars have conducted a meta-analysis and found that systemic antibiotics, as an adjunct to non-surgical periodontal treatment, should be used reasonably and restricted [15]. Because of the increasing demand for periodontal treatment, traditional Chinese medicine has begun to attract people's attention. Articles published in recent years have pointed out that CR has certain anti-inflammatory and osteogenic differentiation properties in the treatment of chronic periodontitis [16–19].

**Fig. 1:**
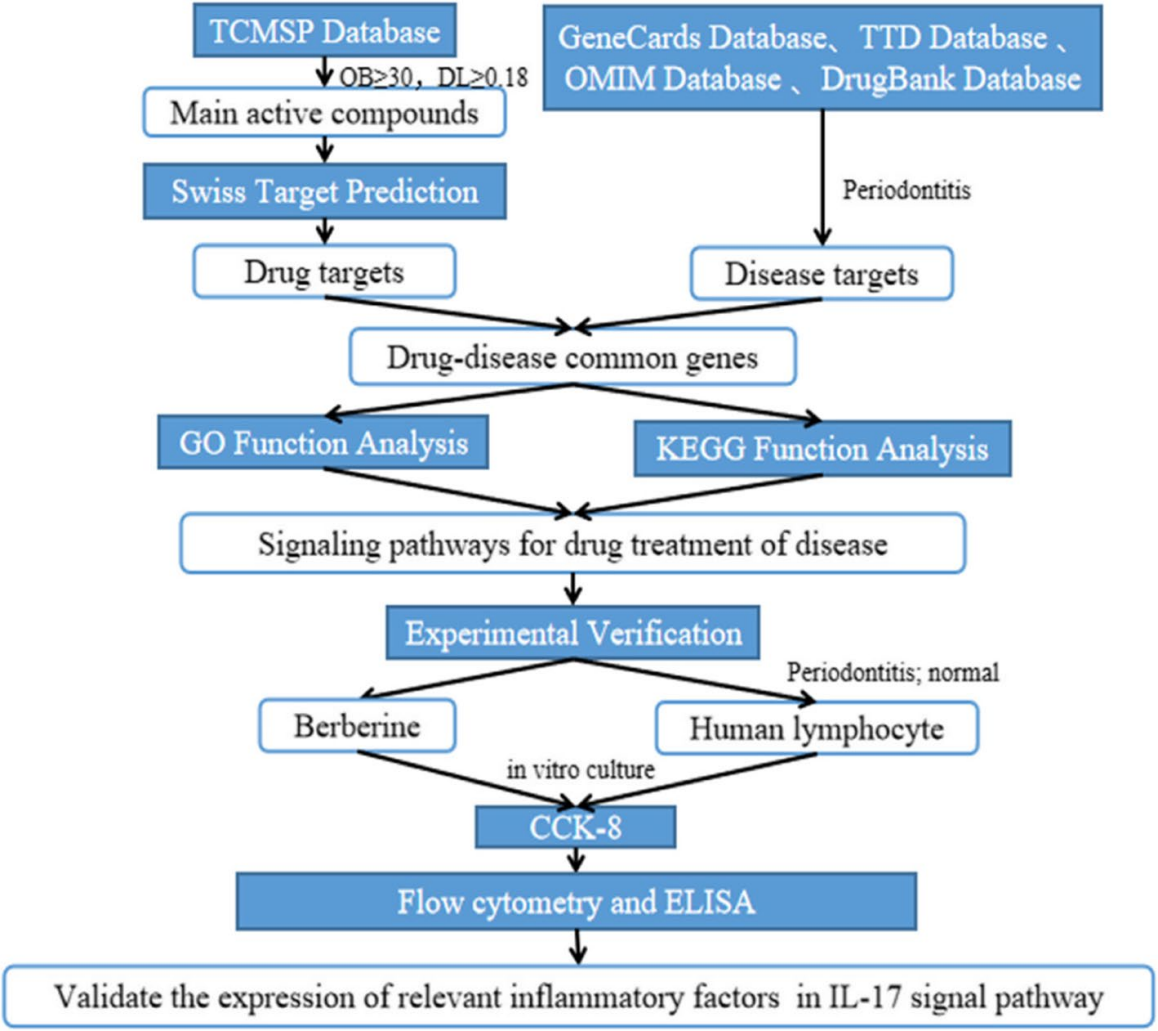
The framework of this study is based on methods in network pharmacology and in vitro experimental verification. TCMSP, Traditional Chinese Medicine Systems Pharmacology; OB, Oral bioavailability; DL, drug similarity; GO, Gene Ontology; KEGG, Kyoto Encyclopedia of Genes and Genomes

Therefore, the purpose of this study was to explore the therapeutic mechanism of CR on periodontitis, and the flowchart is shown in Fig. 1. The relevant targets and signaling pathways of CR in the treatment of periodontitis were preliminarily predicted by the method of network pharmacology. Then use the method of in vitro experiments to verify, and provide scientific basis for further mechanism research.

## Materials and methods

### Section of Network Pharmacology

#### The potential components screening and target prediction of active ingredients of CR

Traditional Chinese Medicine Systems Pharmacology (TCMSP) is a unique pharmacological platform for traditional Chinese medicine systems, where we can understand the relationships between drugs, targets, and diseases(https://old.tcmsp-e.com/tcmsp.php). Using "Huanglian" as the search term, the active chemical constituents of traditional Chinese medicine CR were searched in the TCMSP database. OB ≥ 30%, DL ≥ 0.18 [20, 21] were screened to obtain the potential active ingredients of CR.

The SMILES structures of the potential active components of CR were sequentially entered into the Swiss Target Prediction database (http://swisstargetprediction.ch/), and the “Probility > 0” was used as the screening condition to obtain the predicted targets of CR-related active ingredients.

#### Screening of target genes for periodontitis disease

Genecards(https://www.genecards.org/) is a comprehensive database of searchable genes, where we can obtain information on almost all known human genes. Using "periodontitis" as the search term in GeneCards, TTD (http://db.idrblab.net/ttd/), OMIM (https://omim.org/), DrugBank (https://go.drugbank.com) database was searched for disease-related target genes. Merge the results from the four databases and remove duplicates.

#### Construction of the gene network map of the intersection of CR and periodontitis

Using the Venny 2.1.0 platform (https://bioinfogp.cnb.csic.es/tools/venny/index.html), the drug targets and disease targets predicted by the above screening were crossed to obtain a common target set. These are the potential targets of CR for the treatment of periodontitis. Import the above potential targets into String (https://cn.string-db.org/), and select "Homo sapiens" for analysis. Finally, the analysis results were saved, and the results were visualized in Cytoscape3.7.1 software.

#### GO and KEGG enrichment analysis

The screened common target genes were imported into the Metascape platform (https://metascape.org/gp/index.html), then analyzed and the data was saved. The results were visualized by using the R language package clusterProfiler to obtain the top 10 GO enrichment analysis and KEGG signaling pathway according to the *P* value.

#### Molecular docking

Download the 3D structure of BBR through the Pubchem database, and obtain 30 cross target receptor proteins from the Uniprot database. Obtain the corresponding PDB IDs through the RCSB PDB database to obtain the 3D structure of the proteins. The specific method is to first search for the target protein in the RCSB PDB database, and secondly narrow down the range based on the conditions on the left: (1) What experimental method was used to obtain the protein structure, among which X-ray crystallography is still the main method for obtaining the protein tertiary structure; (2) Resolution: Å is a commonly used unit of measurement for the length of light waves and the diameter of molecules. The smaller the value, the higher the resolution, and the more accurate the structure. Therefore, we prioritize choosing the smaller value; (3) Time to obtain protein structure: Generally speaking, the newer the year, the better. Finally, select the PDB ID of the optimal protein for subsequent experiments. Use Discovery Studio software to prepare ligands for BBR components, and then perform dehydration, ligand removal, and hydrogen supplementation treatments on 30 receptor proteins one by one. Macromolecular docking is performed in the "LibDock" module. Calculate the combination of BBR molecules and 30 protein receptors to sort according to small to large. Generally speaking, the lower the binding, the more stable, and the results of several sets of ligands are visualized. These graphics can display the interaction between the receptor protein and the small molecule of the ligand through the number of hydrogen bonds [22].

### Section of in vitro experiment

#### In vitro experimental materials

##### Cell source

The study is approved by Ethics Committee of Guiyang Dental Hospital (ethical review batch number: GYSKLL-KY-20111210–01). Peripheral blood of healthy people and peripheral blood of patients with chronic periodontitis are provided by volunteer patients and volunteer employees who will be treated in Guiyang Stomatological Hospital, Nanming District, Guiyang City, Guizhou Province from December 2021 to July 2022. All participants provided written informed consent, and the procedure complied with the requirements of the Ethics Committee of Guiyang Stomatological Hospital.

Inclusion criteria: Chinese; adults and about 30–40 years old; at least 20 teeth in the whole mouth that can be evaluated for periodontal evaluation and diagnosed as patients with chronic periodontitis; no smoking history; medical history and physical examination within one year; no immune-related diseases; no orthodontic treatment history; no antibiotics and immunosuppressants within 3 months; no systemic periodontal treatment; no scaling within half a year; women without pregnancy and breastfeeding. Exclusion criteria: After excluding occlusal trauma factors, food impaction factors, and periodontal-pulp combined disease factors, at least one tooth in each quadrant has a periodontal pocket > 4 mm and attachment loss (CAL) ≥ 3 mm [23].

##### Separation of PBMCs

Collect 5 ml of venous blood at the elbow with heparin sodium anticoagulation from each normal volunteer and chronic periodontitis patient, and the lymphocyte separation method is based on the research protocol of Dagur PK et al. [24]. Add equal volume of PBS(company: Biological Industries) to dilute the blood. Ficoll-Hypaque(company: Solarbio) was added to the centrifuge tube and the diluted blood was slowly layered on top. Peripheral blood mononuclear cells (PBMC) were isolated by density gradient centrifugation. Carefully suck out the mononuclear cell layer into a conical tube, add PBS and mix, centrifuge at 1000 rpm for 5 min, discard the supernatant, and repeat twice to obtain the final extraction of PBMC.

##### CCK-8

The main components of CR for experimental medicine: BBR hydrochloride hydrate (Item No.: B107342, purity 98%), purchased from Aladdin Reagent Co., Ltd.

The lymphocyte proliferation experiment was carried out by CCK-8 method, and the optimal LPS concentration and CR concentration were screened out as the subsequent in vitro experimental conditions. PBMCs were suspended in DMEM medium(company: HyClone) containing 10% calf serum(company: Every green) and added to a 96-well plate (about 5000 cells/well). Set up different experimental groups and add different concentration gradients of lipopolysaccharide or BBR solutions to stimulate. The blank group was added with the same volume of 10% calf serum DMEM medium. 4.5 h before the end of each cell culture period, 10μL/well of CCK-8 solution was added and incubated with the cells until the end time. Finally, use a microplate reader to measure the absorbance at 450 nm.

##### Flow cytometry

Flow cytometry is widely used to analyze the expression of molecules on cell surfaces and within cells, identify and determine different cell types in heterogeneous cell populations, evaluate the purity of isolated subpopulations, and analyze cell size and volume. The method of determining T cell subsets refers to the research protocol of Falay M et al. [25]. Take 100ul of anticoagulant from the participants, add 10ul of CD3-FITC/CD8-PE/CD45-PerCP/CD4-APC(company: Agilent) mixed monoclonal fluorescent antibody, and incubate at room temperature for 15 min in the dark. Add 500 μl of red blood cell lysate, mix by gently pipetting, and incubate at room temperature for 10 min in the dark. Add 1 ml of PBS for washing, centrifuge at 1500 rpm for 5 min, and discard the supernatant. Finally, 0.5 ml of PBS was added to resuspend the cells, and the cells were detected and analyzed on the machine.

Collect about 5 × 10^5^ lymphocytes in the experimental group after 72 h of culture, add 100 μl PBS and mix well. Then add 10 μl CD3-FITC/CD8-PE/CD45-PerCP/CD4-APC mixed monoclonal fluorescent antibody and repeat the above steps. After antibody incubation, PBS was added for washing. The stained cells were resuspended in 0.5 ml PBS, and the cells were detected and analyzed on the machine.

Suspend the cells in PBS, adjust the concentration to 1–2 × 10^7^ cells/ml, and add 2 μl of Cell Activation Coctail (with Brefeldin A) per 1 ml of cell suspension. It was spread into a 12-well plate, and 1 ml of serum-free DMEM medium was added to each well, followed by culturing for 6 h in a 37 °C, 5% CO_2_ incubatorCells were harvested and suspended in 100 μl of PBS. Add CD4 antibody(APC anti-human CD4, Clone RPA-T4, company: Biolegend) to each tube of cells and incubate at room temperature for 15 min in the dark. Fix the cells by adding Fixation Buffer(company: Biolegend) and incubate at room temperature for 20 min in the dark. Add 1 ml of 1 × Permeabilization Wash Buffer(company: Biolegend) working solution to each tube, wash and centrifuge. Add 100ul of Permeabilization Wash Buffer to resuspend the cells, then add IL-17 antibody(PE anti-human IL-17A, Clone BL168, company: Biolegend), and incubate for 30 min at room temperature in the dark. Finally, the cells were washed, centrifuged, and the supernatant was discarded. The stained cells were resuspended and fixed in 0.5 ml PBS, and detected and analyzed on the machine.

##### Elisa

The serum of all subjects was collected, sealed and stored in a -80 °C refrigerator for later detection. The lymphocyte culture supernatants stimulated by lipopolysaccharide for 72 h, lipopolysaccharide and CR for 72 h were collected, sealed and stored in a -80 °C refrigerator for later detection. The secretion levels of IL-6, IL-8, IL-17 cytokines in the samples were determined using the relevant ELISA kits according to the manufacturer's instructions(HumanIL-17A ELISA Kit, Human IL-6 ELISA Kit, Human IL-8 ELISA Kit, All purchased from Elabscience, 96 T).

### Statistical analysis

The data were expressed as mean ± SD, and the results were retained to two or three decimal places. The parameters between the two groups were compared by two independent sample t-test, and the parameters between multiple groups were compared by one-way analysis of variance. All data were evaluated by IBM SPSS statistics 20 and graphpad prism V.9 software, *P* < 0.05 was significant, *P* < 0.01 was extremely significant.

## Results

### Section of network pharmacology

#### The potential components screening and target prediction of active ingredients of CR

**Fig. 2:**
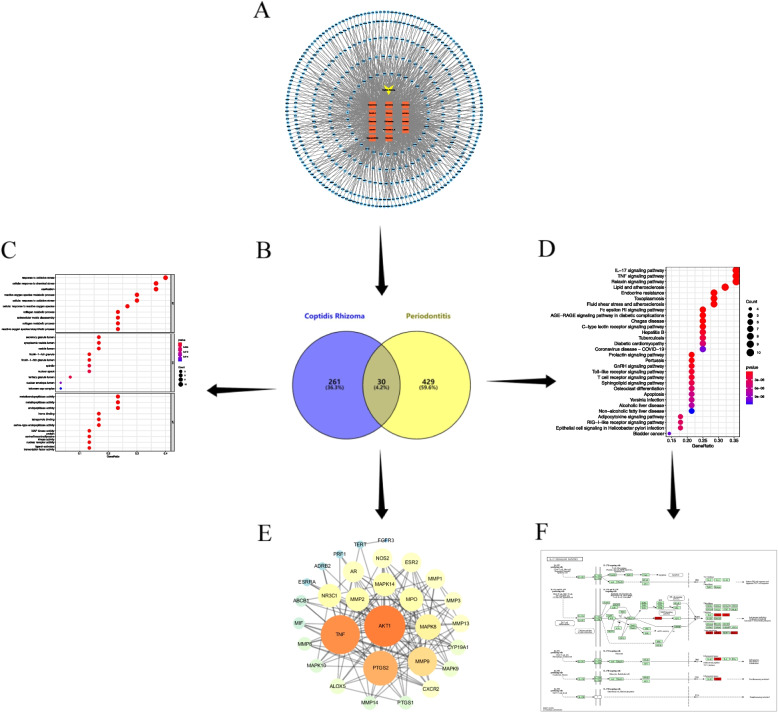
**A** CR, active ingredients and their corresponding target genes; **B** Venn diagram of CR and periodontitis; **C** GO enrichment analysis bubble plot; **D** KEGG enrichment analysis bubble plot; **E** PPI of 30 target genes; **F** IL-17 signaling pathway map, core target genes are marked in red

A total of 48 active ingredients were screened from the TCMSP database, and 14 active ingredients were obtained after further screening using the benchmark conditions of OB ≥ 30% and DL ≥ 0.18. Using the Swiss Target Prediction database, the SMILES structure of the potential active ingredients was used to search, and the "Probility > 0" was screened. After removing the duplicates, a total of 291 potential active ingredients of CR were predicted (Fig. 2A).

#### Screening of target genes for periodontitis disease

The number of periodontitis-related target genes retrieved from the four databases of GeneCards, TTD, OMIM and DrugBank was 8, 34, 2, and 2454. Subsequently, 418 targets in the DrugBank database were selected with "Score ≥ 3" as the screening condition. The results of the four databases were merged and duplicates were removed, and finally a total of 459 target genes for periodontitis were obtained. These 459 genes are the final genes related to periodontitis that we have selected from four comprehensive gene libraries, which provide all the annotations and predictable genes currently available to humans.

#### Construction of the gene network map of the intersection of CR and periodontitis

The action targets of the potential active components of CR and the periodontitis disease targets were merged and then intersected, and finally 30 potential targets were obtained (Fig. 2B). Protein–Protein Interaction Networks (PPI) of the intersection targets of CR and periodontitis were constructed (Fig. 2E). The size and gradient color of each target in the figure are adjusted by Degree. Line thickness and gradient color are adjusted by combined-score.

#### GO and KEGG enrichment analysis

GO enrichment analysis was performed on 30 targets, respectively from biological process (BP), cell component (CC), molecular function (MF), and the analysis results showed the top ten (Fig. 2C). BP mainly involves oxidative stress response, cytochemical stress response and ossification; CC mainly involves secretory granule lumen and cytoplasmic vesicle lumen; MF involves metalloendopeptidase activity and metallopeptidase activity. The KEGG enrichment analysis screened out the top 30 results, where the abscissa GeneRatio represents the percentage (%) of the input genes to the overall genes, the size of the circle represents the number of genes (count), and the color of the circle represents *P*-value (Fig. 2D). The results showed that the targets of CR in the treatment of periodontitis mainly involved the IL-17 signaling pathway (Fig. 2F), the TNF signaling pathway and the Relaxin signaling pathway.

#### Molecular docking

**Fig. 3:**
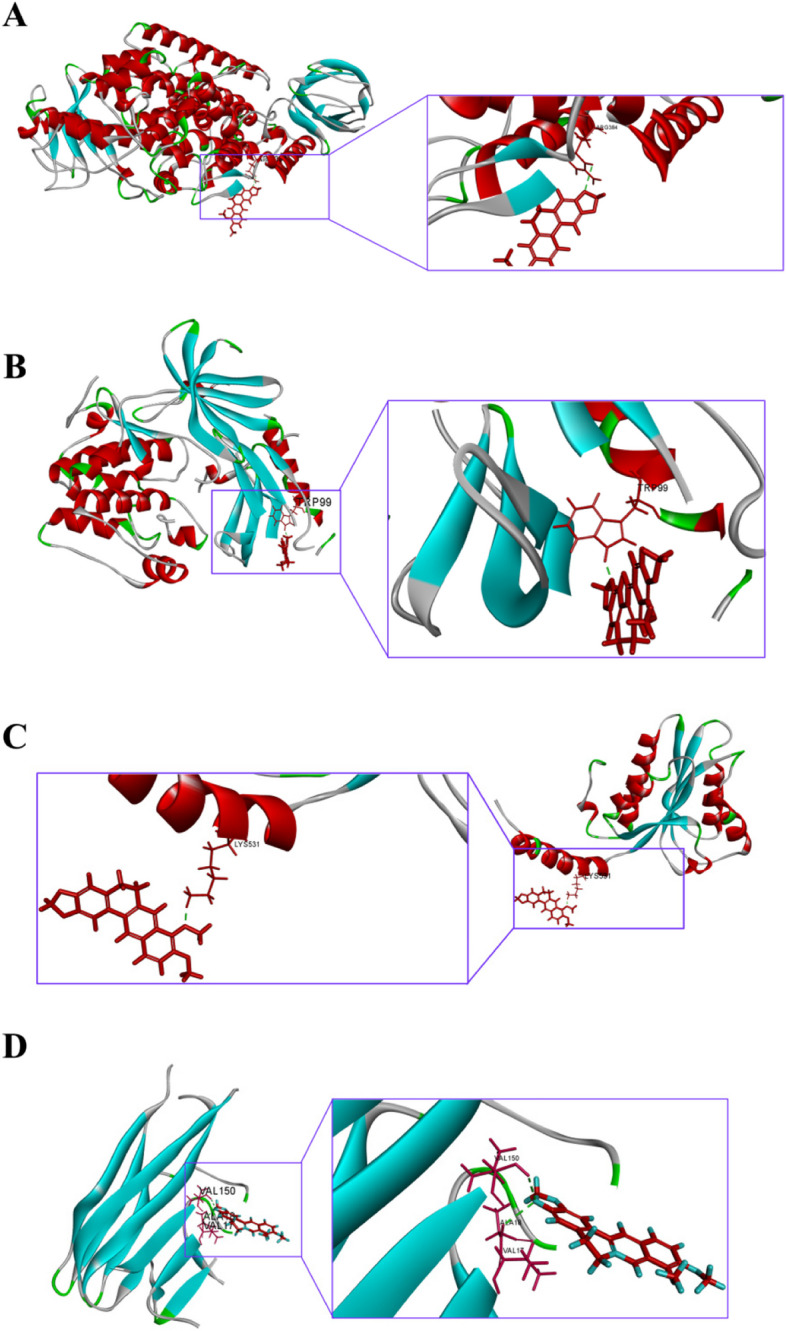
Docking diagram of berberine and target molecule. **A** ALOX5 and Berberine; **B** AKT1 and Berberine; **C** NOS2 and Berberine; **D** TNF and Berberine

The 30 target proteins were successively docked with the BBR for Macromolecular docking, and the sequencing was based on the size of Binding energy. Visualize the docking results between the protein with the lowest energy in the top 4 positions and BBR, which are ALOX5, AKT1, NOS2, and TNF, as shown in Table 1 and Fig. 3. In Table 1, if the Binding energy is lower than 0, the combination effect is better. The lower the energy value, the more stable the combination of the two. The green dashed lines in Figs. 3 represent the hydrogen bonds between BBR and various target proteins. The more hydrogen bonds there are, the more stable their binding is.

**Table 1:** Combination results of ALOX5, AKT1, NOS2, and TNF with BBR

Target	Drug	PDB ID	Binding Energy (Kcal/mol)
ALOX5	BBR	6NCF	-109.27
AKT1	BBR	3O96	-107.61
NOS2	BBR	3HR4	-86.33
TNF	BBR	6X82	-67.34

### Section of In vitro experiment

#### CCK-8

**Fig. 4:**
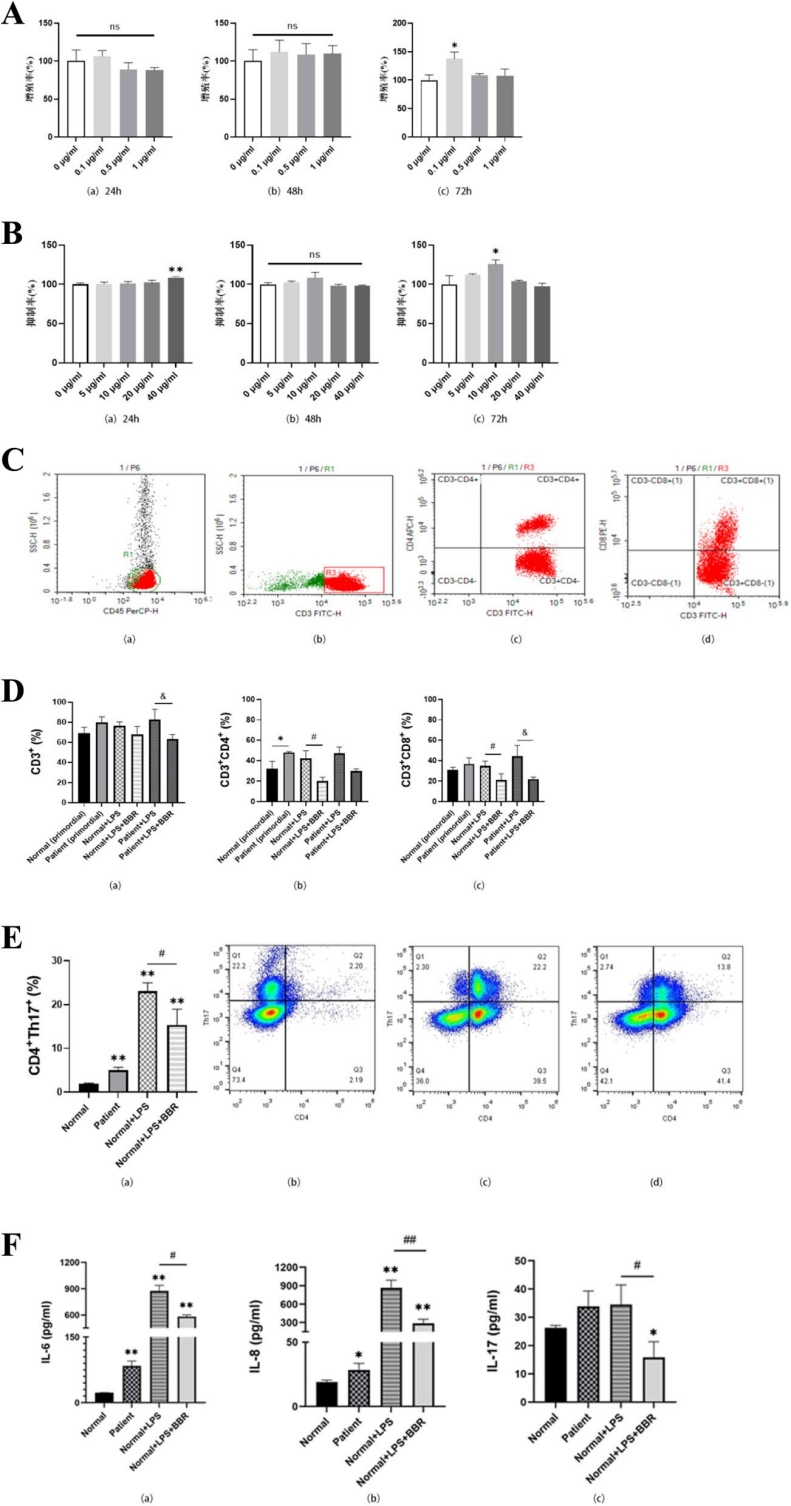
(A) The proliferative effect of LPS at different concentrations on lymphocytes cultured in vitro was detected by CCK-8 method. (a) The proliferation rate of lymphocytes cultured with LPS at a gradient concentration for 24 h; (b) The proliferation rate of lymphocytes cultured with LPS at a gradient concentration for 48 h; (c) The proliferation rate of lymphocytes cultured with LPS at a gradient concentration for 72 h; All values are expressed in mean ± SD, *n* = 3, and compared with the 0 μg/ml LPS group, *P < 0.05, ns: no statistical significance. (B) The inhibitory effect of BBR at different concentrations on lymphocytes intervened in vitro was detected by CCK-8 method. (a) 0.1 μg/ml LPS + BBR with gradient concentration jointly intervenes the proliferation rate after 24 h of lymphocytes; (b) 0.1 μg/ml LPS + BBR with gradient concentration jointly intervenes the proliferation rate after 48 h of lymphocytes; (c) 0.1 μg/ml LPS + BBR with gradient concentration jointly intervenes the proliferation rate after 72 h of lymphocytes; All values are expressed in mean ± SD, *n* = 3, and compared with the 0 μg/ml BBR + 0.1 μg/ml LPS group, * *P* < 0.05, **P < 0.01. (C) Fig. 4–4 Flow cytometry of lymphocyte subpopulations. (a) The expression level of CD45 in PBMC cells; (b) The expression level of CD3; (c) The expression level of CD3 + and CD4 + ; (d) The expression level of CD3 + CD8 + . (D) Histogram of expression of lymphocyte subsets. (a) The expression of CD3 + in each group; (b) The expression of CD3 + CD4 + in each group; (c) The expression of CD3 + CD8 + in each group; All values are expressed in mean ± SD, *n* = 3; Compared with Normal (primordial) group, *P < 0.05; Compared with Normal + LPS group, #*P* < 0.05; Compared with Patient + LPS group, &*P* < 0.05. (E) Expression of CD4 + Th17 + . (a) Histogram of CD4 + Th17 + expression; (b) original in normal group; (c) normal group + LPS; (d) normal group + LPS + BBR; All values are expressed in mean ± SD, *n* = 3;Compared with the Normal group, **P < 0.01; Compared with Normal + LPS group, #*P* < 0.05. (F) Standard curve of inflammatory factors and histogram of their expression. (a) IL-6 expression; (b) IL-8 expression; (c) IL-17 expression; All values are expressed in mean ± SD, *n* = 3; Compared with the Normal group, *P < 0.05, **P < 0.01; Compared with Normal + LPS group, #*P* < 0.05, ##*P* < 0.01

The final results of the CCK-8 method are shown in Fig. 4A, Using with 0 μg/ml LPS for each group at 24, 48, and 72 h as the blank control group and LPS with different concentration gradients as the experimental group, the proliferation rate was calculated based on the measured OD values. The proliferation rate of the 0.1 μg/ml LPS group was higher than that of the control group (0 μg/ml LPS group) was relatively high after 72 h of intervention, and the difference was statistically significant (*P* < 0.05). The differences in the other time periods were not statistically significant, so 0.1μg/ml LPS was chosen was used as an in vitro intervention experimental condition for subsequent lymphocytes. Add 0.1μg/ml LPS to each group stimulated lymphocytes, and different concentration gradients of BBR were added to the experimental group for intervention, with the 0 μg/ml BBR group was used as a blank control, and the inhibition rate was calculated based on the OD value, as shown in Fig. 4B. During these three time periods, after 24 h of intervention with add 40 μg/ml BBR on lymphocytes, the inhibition rate was higher than that of the blank group, and there was a statistical difference (*P* < 0.01). There was no significant difference in the inhibition rate after 48 h of intervention with gradient concentration BBR compared to the blank control group at the same time. When after 72 h of intervention with adding 10 μg/ml BBR, the inhibition rate was significantly higher than that of the blank group (*P* < 0.05). Finally, based on the principles of economy and timeliness of the experiment, 10μg/ml BBR was selected for subsequent experiments as the intervention concentration.

#### Flow cytometry

According to the detection results of CCK-8, the concentration of LPS and BBR was selected for 0.1 μg/ml and 10 μg/ml in subsequent experiments. After 72 h of intervention, the next detection was performed. After 72 h of lymphocyte intervention, flow cytometry was performed according to the method in the steps. The expression levels of initial CD3 + , CD4 + and CD8 + in peripheral blood lymphocytes of the normal group and the patient group were analyzed, as well as the expression changes of CD3 + , CD4 + and CD8 + in peripheral blood lymphocytes of the normal group and the patient group after the intervention in vitro. The results are shown in Fig. 4C, D. In Fig. 4D-a, the proportion of CD3 + in the patient group + LPS was higher than that in the patient group + LPS + BBR (*P* < 0.05), indicating that adding BBR intervention can reduce the proportion of CD3 + in lymphocytes stimulated by LPS. In Fig. 4D-b, the CD4 + expression in the normal group was lower than that in the patient group, and was statistically significant. The expression of CD4 + in the normal group + BBR + LPS was lower than that in the normal group + LPS, and there were differences. Similarly, in Fig. 4D-c, the proportion of CD8 + in the normal group and the patient group after the intervention with BBR + LPS was lower than that in the normal group and the patient group after the intervention with LPS only, and there were differences.

Add 0.1 μg/ml LPS and 10 μg/ml BBR co intervened with lymphocytes for 72 h, and then flow cytometry was used to analyze the changes in the proportion of CD4 + Th17 + , as shown in Fig. 4E. The comparison of CD4 + Th17 + expression levels in lymphocytes of the normal group stimulated by drugs is shown in Fig. 4E-b,c,d, where the Q2 region represents the proportional value of CD4 + Th17 + . In Fig. 4Ea, with the normal group as the control, the proportion of CD4 + Th17 + in the other three groups significantly increased (*P* < 0.01). The proportion of CD4 + Th17 + in the normal group + LPS + BBR decreased compared to the normal group + LPS, and there was a difference (*P* < 0.05). This result indicates that the proportion of CD4 + Th17 + in patients is higher than that in normal individuals, and BBR reduces the proportion of CD4 + Th17 + stimulated by LPS in lymphocytes.

#### Elisa

The R^2^ of the standard curve determined by Elisa reagent used in this experiment is ≈ 0.999. Collect normal's serum, patient's serum, and supernatant after 72 h of intervention lymphocytes, and measure the expression levels of IL-6, IL-8, and IL-17 inflammatory factors in serum and supernatant using Elisa reagent kit, as shown in Fig. 4F. The results showed that, with the normal group as the control, the levels of IL-6 and IL-8 in the serum of the patient group increased, and there was a difference (*P* < 0.05). After adding LPS to the normal group, the expression levels of IL-6 and IL-8 increased (*P* < 0.01). The expression levels of IL-6, IL-8, and IL-17 in the normal group + LPS + BBR were significantly different from those in the control group (*P* < 0.05). The expression levels of various inflammations in the normal group + LPS + BBR were significantly lower than those in the normal group + LPS (*P* < 0.05). This result indicates that the three inflammatory factors in the patient's serum are higher than those in normal's serum, and BBR can effectively reduce the levels of IL-6, IL-8, and IL-17 stimulated by LPS in lymphocytes.

## Discussion

With the rapid development of bioinformatics, systems biology, and polypharmacology, network pharmacology is considered as a promising approach for more cost-effective drug development [26–28]. Chinese herbal medicine has rich experience in clinical treatment, but it has not been recognized by most researchers in international medicine. Therefore, this study links the emerging network science with traditional Chinese medicine, in order to reveal the mechanism of traditional Chinese medicine in treating diseases, and at the same time, it is hoped that the method based on in vitro experiments can provide some scientific evidence for traditional Chinese medicine. In this study, 30 potential targets of CR in the treatment of periodontitis were screened for enrichment analysis, and the KEGG analysis results indicated that the interleukin-17 signaling pathway had the most potential. The molecular docking results showed that BBR can effectively bind to periodontitis targets ALOX5, AKT1, NOS2, and TNF, indicating that these four targets have high biological activity and can serve as potential biomarkers and key therapeutic targets for BBR treatment of periodontitis. Therefore, relevant in vitro validation experiments were designed according to the results of IL-17 signaling pathway screened by the network. The drug selected the Active ingredient BBR with the largest content in CR, and isolated lymphocytes from the peripheral blood of normal people and patients for drug intervention detection and comparison. Experiments have found that BBR can reduce the expression of CD3 + , CD4 + , CD8 + , and Th17 + in lymphocytes, as well as the expression of IL-6, IL-8, and IL-17 inflammatory factors, playing an anti-inflammatory role in the treatment of periodontitis.

In 2000, the active ingredient compounds in CR were extracted by Japanese scholar Hu et al. [29], and the results showed that the extract had obvious inhibitory effect on Porphyromonas gingivalis and other common bacteria in the oral cavity, but the specific effect was the effective antibacterial mechanism pathway has not been clearly studied. Therefore, this study conducted a preliminary bioinformatics analysis of the mechanism and pathway of BBR treatment for periodontitis based on network pharmacology methods. In our bioinformatics results, we found that the IL-17 signaling pathway plays a crucial role in the treatment of periodontitis. After consulting literature, it was found that there are also many studies that can prove the relationship. Luo and Nair et al. found that the mRNA levels of IL-17A and IL-17F were different in the gingival tissue of healthy people and patients with chronic periodontitis, and the expression was higher in patients [30, 31]. Mohammadian et al. explored the anti-inflammatory effect of BBR in periodontitis from in vitro and in vivo experimental models [16]. It was found that BBR exerts an anti-inflammatory effect by reducing the expression and secretion of multiple pro-inflammatory mediators such as TNF-α, IL-1β, and IL-17, thereby inhibiting the destruction of periodontal tissue and alveolar bone. These studies have proved that IL-17 plays a non-negligible role in the mechanism of action in the treatment of periodontitis. A study has found that the triggering factor for the excessive accumulation of Th17 cells in healthy gums is sustained damage during chewing [32]. Because injury can induce epithelial cells to produce IL-6, thereby promoting an increase in the number of gingival Th17 cells in an antigen-dependent manner. Th17 has a great relationship with periodontitis, and Th17 balance regulation is key in the treatment of periodontitis. Based on all published research, the bioinformatics analysis results of this study are further confirmed.

It is undeniable that glucocorticoids, Nonsteroidal anti-inflammatory drug, antibiotics and so on are the first-line drugs in the traditional treatment of immune mediated inflammatory diseases. However, intolerance and severe adverse reactions have increased the demand for alternative therapies, such as the repair effect of traditional Chinese medicine on the intestinal mucosal barrier after oral administration of Western medicine [33]. Therefore, this study chose traditional Chinese medicine for relevant research. Chinese people use traditional herbal formulas to treat diseases, among which CR is more commonly used. It has the effects of clearing heat, resolving moisture, purifying fire, and detoxifying. It also plays a role in the oral cavity. Takuma Okuda et al. found that BBR reduces the relative abundance of periodontal pathogens in the oral microbiome, has antibacterial and anti-inflammatory effects, and can help prevent periodontal diseases [34]. Other studies have found that BBR can repair bone destruction in periapical disease by reducing the expression of metalloproteinases and increasing the expression of lysyl oxidase [35–37]. Ying Zhang et al. used metabonomics to study BBR in oral cavity. The results showed that BBR could inhibit inflammatory factors (IL-6, IL-1 β and TNF- α) in human gingival fibroblasts and LPS induced apoptosis signal pathway [38]. A study found that BBR can be induced by the expression of β-catin can up regulate the protein expression of Runx2, and promote the osteogenic differentiation of root tip stem cells [17]. These studies are also consistent with the in vitro experimental results of this study, further verifying the credibility of the results.

Chinese herbal medicine has rich clinical treatment experience, but it has not been recognized by most researchers in the international medical community. Due to its complex components, the mechanism of action is intricate and complex, and multiple components can interact with each other. Therefore, the future research prospects of Chinese herbal medicine still face significant challenges to overcome. Therefore, this study will link the emerging network science with traditional Chinese medicine, in order to reveal the mechanism of traditional Chinese medicine in the treatment of diseases, and hope that the method based on in vitro experiment can provide some scientific basis for traditional Chinese medicine.

## Conclusions

This study used network pharmacology to explore the therapeutic effect of CR in periodontitis, and then used molecular docking experiment and in vitro experiment to verify that BBR, the most effective chemical component in CR, can treat periodontitis through IL-17 signaling pathway and has anti-inflammatory effect. This study provides new ideas, methods, and theoretical basis for the use of traditional Chinese medicine in the treatment of periodontitis.

## Data Availability

Data is provided within the manuscript or supplementary information files.

## References

[1] Wang J, Wang L, Lou GH, Zeng HR, Hu J, Huang QW, Peng W, Yang XB (2019). Coptidis Rhizoma: a comprehensive review of its traditional uses, botany, phytochemistry, pharmacology and toxicology. Pharm Biol.

[2] Tan L, Li C, Chen H, Mo Z, Zhou J, Liu Y, Ma Z, Xu Y, Yang X, Xie J (2017). Epiberberine, a natural protoberberine alkaloid, inhibits urease of Helicobacter pylori and jack bean: Susceptibility and mechanism. Eur J Pharm Sci.

[3] Wang H, Li K, Ma L, Wu S, Hu J, Yan H, Jiang J, Li Y (2017). Berberine inhibits enterovirus 71 replication by downregulating the MEK/ERK signaling pathway and autophagy. Virology journal.

[4] Kim SY, Park C, Kim MY, Ji SY, Hwangbo H, Lee H, Hong SH, Han MH, Jeong JW, Kim GY (2021). ROS-mediated anti-tumor effect of Coptidis Rhizoma against human hepatocellular carcinoma Hep3B cells and xenografts. Int J Mol Sci.

[5] Chakravarthy D, Muñoz AR, Su A, Hwang RF, Keppler BR, Chan DE, Halff G, Ghosh R, Kumar AP (2018). Palmatine suppresses glutamine-mediated interaction between pancreatic cancer and stellate cells through simultaneous inhibition of survivin and COL1A1. Cancer Lett.

[6] Ran Q, Wang J, Wang L, Zeng HR, Yang XB, Huang QW (2019). Rhizoma coptidis as a potential treatment agent for type 2 diabetes mellitus and the underlying mechanisms: a review. Front Pharmacol.

[7] Wang Z, Yang Y, Liu M, Wei Y, Liu J, Pei H, Li H (2020). Rhizoma Coptidis for Alzheimer's disease and vascular dementia: a literature review. Curr Vasc Pharmacol.

[8] Li S, Hao L, Deng J, Zhang J, Hu X (2023). Coptidis rhizoma and evodiae fructus against lipid droplet deposition in nonalcoholic fatty liver disease-related liver cancer by AKT. Chem Biol Drug Des.

[9] Al-Kanaan BM, Al-Ouqaili MTS, Al-Rawi KFA (2019). Detection of cytokines (IL-1α and IL-2) and oxidative stress markers in hepatitis B envelope antigen-positive and -negative chronic hepatitis B patients: Molecular and biochemical study. Gene Reports.

[10] Renvert S, Persson GR (2016). Treatment of periodontal disease in older adults. Periodontology 2000.

[11] Aldelaimi AAK, Enezei HH, Aldelaimi TN, Mohammed KA (2021). Tumors of Craniofacial Region in Iraq (Clinicopathological Study). J Res Med Dent Sci..

[12] Aldelaimi TN, Khalil AA (2015). Clinical application of diode laser (980 nm) in Maxillofacial surgical procedures. J Craniofac Surg.

[13] Takallu S, Mirzaei E, ZakeriBazmandeh A, GhaderiJafarbeigloo HR, Khorshidi H (2024). Addressing antimicrobial properties in guided tissue/bone regeneration membrane: enhancing effectiveness in periodontitis treatment. ACS Infect Dis.

[14] Tao S, Chen H, Li N, Wang T, Liang W (2022). The spread of antibiotic resistance genes in vivo model. Can J Infect Dis Med Microbiol.

[15] Pretzl B, Sälzer S, Ehmke B, Schlagenhauf U, Dannewitz B, Dommisch H, Eickholz P, Jockel-Schneider Y (2019). Administration of systemic antibiotics during non-surgical periodontal therapy-a consensus report. Clin Oral Investig.

[16] Mohammadian Haftcheshmeh S, Momtazi-Borojeni AA (2021). Berberine as a promising natural compound for the treatment of periodontal disease: a focus on anti-inflammatory properties. J Cell Mol Med.

[17] Cui Y, Xie J, Fu Y, Li C, Zheng L, Huang D, Zhou C, Sun J, Zhou X (2020). Berberine mediates root remodeling in an immature tooth with apical periodontitis by regulating stem cells from apical papilla differentiation. Int J Oral Sci.

[18] Liu J, Zhao X, Pei D, Sun G, Li Y, Zhu C, Qiang C, Sun J, Shi J, Dong Y (2018). The promotion function of Berberine for osteogenic differentiation of human periodontal ligament stem cells via ERK-FOS pathway mediated by EGFR. Sci Rep.

[19] Zhang LN, Wang XX, Wang Z, Li KY, Xu BH, Zhang J (2019). Berberine improves advanced glycation end products-induced osteogenic differentiation responses in human periodontal ligament stem cells through the canonical Wnt/β-catenin pathway. Mol Med Rep.

[20] Xiang L, Bo X, Xin L, Jiang XW, Lu HY, Xu ZH, Yue Y, Qiong W, Dong Y, Zhang YS (2021). Network pharmacology-based research uncovers cold resistance and thermogenesis mechanism of Cinnamomum cassia. Fitoterapia.

[21] Xiong H, Dong Z, Lou G, Gan Q, Wang J, Huang Q (2020). Analysis of the mechanism of Shufeng Jiedu capsule prevention and treatment for COVID-19 by network pharmacology tools. Eur J Integ Med.

[22] Jofily P, Pascutti PG, Torres PHM (2021). Improving blind docking in DOCK6 through an automated preliminary fragment probing strategy. Molecules..

[23] Armitage GC (2000). Periodontal diagnoses and classification of periodontal diseases. Periodontol.

[24] Dagur PK, McCoy JP (2015). Collection, storage, and preparation of human blood cells. Curr Protoc Cytom.

[25] Falay M, Senes M, Korkmaz S, Zararsız G, Turhan T, Okay M, Yücel Ç, Kılınckaya MF, Ozet G, Yucel D (2019). Biological variation of peripheral blood T-lymphocytes. J Immunol Methods.

[26] Zhang R, Zhu X, Bai H, Ning K (2019). Network pharmacology databases for traditional Chinese medicine: review and assessment. Front Pharmacol.

[27] Zheng S, Xue T, Wang B, Guo H, Liu Q (2022). Application of network pharmacology in the study of the mechanism of action of traditional chinese medicine in the treatment of COVID-19. Front Pharmacol.

[28] Yang MQ, Chen C, Mao YF, Li Y, Zhong X, Yu YD, Xue YT, Song YM (2022). Application of network pharmacology and molecular docking approach to explore active compounds and potential pharmacological mechanisms of Aconiti Lateralis Radix Praeparata and Lepidii Semen Descurainiae Semen for treatment of heart failure. Medicine.

[29] Hu JP, Takahashi N, Yamada T (2000). Coptidis rhizoma inhibits growth and proteases of oral bacteria. Oral Dis.

[30] Luo Z, Wang H, Chen J, Kang J, Sun Z, Wu Y (2015). Overexpression and potential regulatory role of IL-17F in pathogenesis of chronic periodontitis. Inflammation.

[31] Nair V, Grover V, Arora S, Das G, Ahmad I, Ohri A, Sainudeen S, Saluja P, Saha A (2022). Comparative evaluation of gingival crevicular fluid interleukin-17, 18 and 21 in different stages of periodontal health and disease. Medicina (Kaunas).

[32] Dutzan N, Abusleme L, Bridgeman H, Greenwell-Wild T, Zangerle-Murray T, Fife ME, Bouladoux N, Linley H, Brenchley L, Wemyss K (2017). On-going mechanical damage from mastication drives homeostatic Th17 cell responses at the oral barrier. Immunity.

[33] Che Q, Luo T, Shi J, He Y, Xu DL (2022). Mechanisms by which traditional Chinese medicines influence the intestinal flora and intestinal barrier. Front Cell Infect Microbiol.

[34] Okuda T, Jo R, Tsutsumi K, Watai D, Ishihara C, Yama K, Aita Y, Inokuchi T, Kimura M, Chikazawa T, Nishinaga E, Yamamoto K. An in vitro study of the effects of Phellodendron bark extract and berberine chloride on periodontal pathogenic bacteria in the oral microbiome. J Oral Biosci. 2023 Mar;65(1):72-79. 10.1016/j.job.2022.11.003 36473619

[35] Cui Y, Xie J, Cai L, Zhang D, Sun J, Zhou X. Berberine regulates bone metabolism in apical periodontitis by remodelling the extracellular matrix. Oral Dis. 2023 Apr;29(3):1184-96. 10.1111/odi.14094 34874590

[36] Al-Ouqaili MT. Molecular and genotypic study of SENV-D virus coinfection in Î^2^-thalassemic patients infected with the hepatitis C virus in Iraq. Int J Green Pharm. 2018;12(4):S926-936.

[37] Al-ouqaili MTS. Depending on HPLC and PCR, detection of aflatoxin B1 extracted from Aspergillus flavus strains and it’s cytotoxic effect on AFB treated-hematopoietic stem cells obtained from human umbilical cord. Asian J Pharm. 2018;12(3):S1048-S1054.

[38] Zhang Y, Guo Y, Wei W, Zhang Z, Xu X (2022). Metabolomics profiling reveals berberine-inhibited inflammatory response in human gingival fibroblasts by regulating the LPS-induced apoptosis signaling pathway. Front Pharmacol.

